# The Neuroprotective Role of Retbindin, a Metabolic Regulator in the Neural Retina

**DOI:** 10.3389/fphar.2022.919667

**Published:** 2022-07-06

**Authors:** Xue Zhao, Lars Tebbe, Muna I. Naash, Muayyad R. Al-Ubaidi

**Affiliations:** Department of Biomedical Engineering, University of Houston, Houston, TX, United States

**Keywords:** retbindin, neuroprotection, retinal metabolism, retinal regeneration, riboflavin, flavins

## Abstract

Dysregulation of retinal metabolism is emerging as one of the major reasons for many inherited retinal diseases (IRDs), a leading cause of blindness worldwide. Thus, the identification of a common regulator that can preserve or revert the metabolic ecosystem to homeostasis is a key step in developing a treatment for different forms of IRDs. Riboflavin (RF) and its derivatives (flavins), flavin mononucleotide (FMN) and flavin adenine dinucleotide (FAD), are essential cofactors for a wide range of cellular metabolic processes; hence, they are particularly critical in highly metabolically active tissues such as the retina. Patients with RF deficiency (ariboflavinosis) often display poor photosensitivity resulting in impaired low-light vision. We have identified a novel retina-specific RF binding protein called retbindin (Rtbdn), which plays a key role in retaining flavin levels in the neural retina. This role is mediated by its specific localization at the interface between the neural retina and retinal pigment epithelium (RPE), which is essential for metabolite and nutrient exchange. As a consequence of this vital function, Rtbdn’s role in flavin utilization and metabolism in retinal degeneration is discussed. The principal findings are that Rtbdn helps maintain high levels of retinal flavins, and its ablation leads to an early-onset retinal metabolic dysregulation, followed by progressive degeneration of rod and cone photoreceptors. Lack of Rtbdn reduces flavin levels, forcing the neural retina to repurpose glucose to reduce the production of free radicals during ATP production. This leads to metabolic breakdown followed by retinal degeneration. Assessment of the role of Rtbdn in several preclinical retinal disease models revealed upregulation of its levels by several folds prior to and during the degenerative process. Ablation of Rtbdn in these models accelerated the rate of retinal degeneration. In agreement with these *in vivo* studies, we have also demonstrated that Rtbdn protects immortalized cone photoreceptor cells (661W cells) from light damage *in vitro*. This indicates that Rtbdn plays a neuroprotective role during retinal degeneration. Herein, we discussed the specific function of Rtbdn and its neuroprotective role in retinal metabolic homeostasis and its role in maintaining retinal health.

## 1 Introduction

Inherited retinal diseases (IRDs) represent a highly variable group of blinding retinal diseases, which are known to be caused by mutations in more than 300 different genes or loci (https://sph.uth.edu/retnet/). The two most commonly mutated genes in IRD patients are the rod visual pigment rhodopsin (RHO) and peripherin 2 (PRPH2, also known as RDS), a retinal-specific tetraspanin protein that is vital for the structure of the photoreceptor outer segment (OS) (Conley and Naash, 2009; Stuck et al., 2016). To investigate how mutations in photoreceptor-specific genes lead to cell death, various animal models expressing mutant versions of these genes have been generated, and the retinal phenotype in many of these models mimicked human retinal diseases (Collin et al., 2020). Although most retinal phenotypes in these models are associated with photoreceptor degeneration, deciphering the mechanism(s) that lead to photoreceptor cell death in most of these models has been elusive. In some cases, patients are born expressing the mutant proteins, yet the symptoms do not develop until the fourth or the fifth decade of life. In addition, in order to treat any of these inherited diseases, the development of specific gene therapy, viral or non-viral, must be designed for the specific mutation. Thus, it became clear that there is an imminent need for a pan-gene-mutation therapy, which galvanized investigations into disease mechanisms beyond the mutant proteins. As a result, in recent years, our research has focused on the metabolic changes caused by the absence of the native protein or the expression of a mutant protein (for example, Rtbdn^−/−^ (Kelley et al., 2017; Sinha et al., 2021), Rd1 (Murenu et al., 2021), mutations in Prph2 (Ding et al., 2004; Conley et al., 2014; Stuck et al., 2014; Stuck et al., 2016), or mutant Prph2/Rtbdn^−/−^ (Genc et al., 2020a; Genc et al., 2020b). After all, metabolism is the source of energy that cells need to achieve homeostasis or to execute cell death and to provide the metabolic intermediates needed for anabolic activities. More and more data are emerging to support the conclusion that metabolic dysregulations are the main common events in initiating IRDs (Punzo et al., 2012; Griciuc et al., 2014; Joyal et al., 2016; Kaplan et al., 2021; Sinha et al., 2021). Thus, identifying common metabolic regulators involved in retinal degeneration that can preserve or revert the metabolic ecosystem to homeostasis is essential for the future development of neuroprotective therapeutic strategies. Due to the complicated and energy-intensive nature of phototransduction, photoreceptors have higher energy demands than any other cell type (Hurley, 2021), as demonstrated by the retina’s high consumption of glucose and oxygen (Chertov et al., 2011; Damsgaard and Country, 2022).

Nutrient availability and metabolic alterations are likely common mechanisms that play roles in retinal degeneration (Du et al., 2015). The diseased retinas may reprogram their metabolism at any time, employing different energy sources or mechanisms before or after the onset of degeneration in order to adapt to stress and changes to homeostasis. As a result, metabolic failure may ensue, eventually leading to degeneration (Sinha et al., 2021). Improper levels of neuroprotective factors can contribute to the degenerative process in the retina. The negative effects of downregulation of these neuroprotective factors may eventually prevail, leading to retinal degeneration (Ly et al., 2016). Harder et al. reported the idea that a decline in retinal pyruvate levels resulting from intraocular pressure changes is linked to dysregulated metabolism of glucose (Harder et al., 2020). This highlights the importance of investigating retinal metabolism to identify essential players involved in maintaining health or in exacerbating disease mechanisms and developing strategies for their use as common neuroprotective therapeutic targets for IRD cases.

## 2 Retbindin: A Novel Retina Specific Riboflavin-Binding Protein Regulates Flavin Homeostasis in the Retina

**FIGURE 1:**
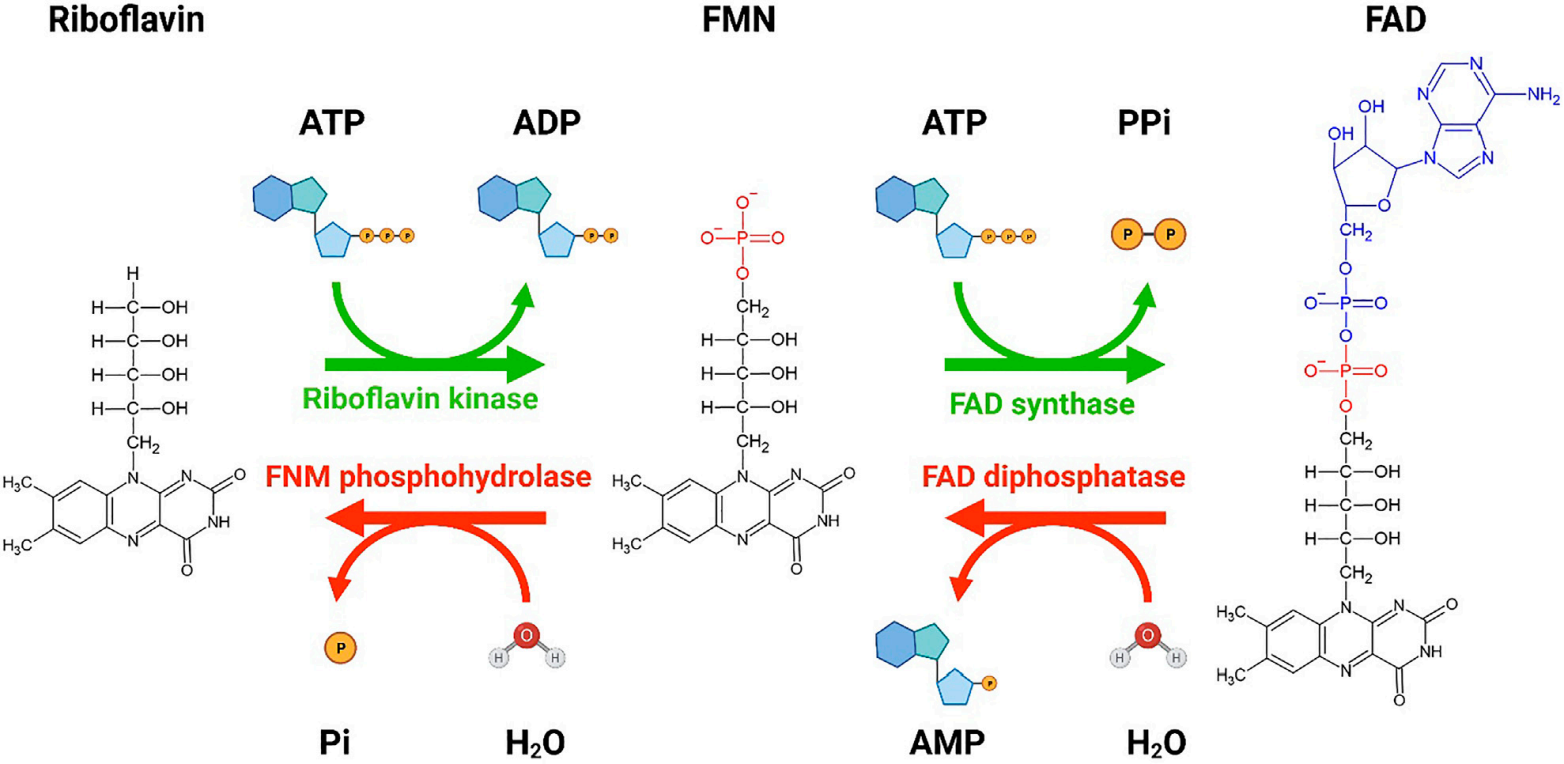
A schematic showing the conversion of riboflavin to flavin mononucleotide (FMN) and flavin adenine dinucleotide (FAD). Bio Render was used to generate the schematic diagram showing the conversion of riboflavin to FMN and FAD by riboflavin kinase and FAD synthase, respectively. Two molecules of ATP are utilized for the conversion of riboflavin to FAD. The reverse conversion of FAD to FMN by FAD diphosphatase and FMN to riboflavin by FMN phosphohydrolase is also shown. The forward conversions are more favorable than the reverse conversion. Re-drawn from Tolomeo et al. (2020). Created with BioRender.

In our approach to investigate the metabolic dysregulation in IRDs, we decided to study pan-metabolic effects rather than focusing on a specific metabolite or pathway. The retinal pigment epithelium (RPE) supplies the photoreceptor cells with essential metabolites by transferring nutrients and oxygen from the choroidal blood supply to the neural retina (reviewed by Sparrow et al. (2010) and Hurley (2021)). Among these metabolites is riboflavin (RF, C_17_H_20_N_4_O_6_), the parent molecule for the cofactors, flavin mononucleotide (FMN, C_17_H_21_N_4_O_9_P), and flavin adenine dinucleotide (FAD, C_27_H_33_N_9_O_15_P_2_) (Figure 1). FMN and FAD, two functionally important cofactors in many redox reactions in metabolism (Powers, 2003), are substantially concentrated in the retina when compared to the blood (Batey and Eckhert, 1990; Batey and Eckhert, 1991; Sinha et al., 2018). RF and its derivatives are involved in various biological processes, including DNA repair, energy metabolism, amino acid synthesis, protein folding, and normal immunological function (Bro-Rasmussen and Horwitt, 1967; Tu et al., 2000; Mazur-Bialy et al., 2013). RF is also an antioxidant, and its absence causes oxidative damage and upregulates stress response in the cell (reviewed by Olfat et al. (2022)). Some of the symptoms seen in individuals lacking RF include growth retardation, hair loss, anemia, nerve impairments, hearing loss, and visual abnormalities [reviewed by Cali et al. (1993) and Mosegaard et al. (2020)]. Ocular abnormalities in humans receiving insufficient RF include corneal lesions, cataract formation, and retinal ganglion cell degeneration, thus, highlighting RF as an essential vitamin in retinal health and survival (Irinoda and Sato, 1954; Venkataswamy, 1967).

The neural retina’s high flavin content suggests that it harbors a unique mechanism for flavin transport/concentration, but until recently, no such systems have been described. Unbound RF can be reduced by light and produce oxygen radicals which lead to peroxidation of lipids in the photoreceptor OS membranes (Oster et al., 1962; Treadwell et al., 1968; Wiegand et al., 1983; Huvaere et al., 2010). As a result, special proteins (flavoproteins) likely play an important role in the sequestration and utilization of flavins in the retina. During our studies on retinal metabolic homeostasis, we became interested in a novel retina-specific RF-binding protein called retbindin (Rtbdn), which has homology to the chicken RF-binding protein (RBP) (Wistow et al., 2002; Kelley et al., 2015). Rtbdn was identified as an expressed sequence tag in the human retina, which Wistow et al. named retbindin due to its retina specificity and the fact that it shares 27% sequence identity (over 135 residues) with RBP (Wistow et al., 2002). Rtbdn is a ∼30 kDa protein that is only found in the neural retina and not in the RPE (Kelley et al., 2017). Rtbdn is located at the junction between the RPE and the photoreceptor OS tips (Kelley et al., 2015). Due to the high levels of flavin in the retina (Batey and Eckhert, 1990; Batey et al., 1992; Sinha et al., 2018) and the fact that the retina has high metabolic activities [reviewed by Country (2017) and Hurley (2021)], we became interested in studying the role of Rtbdn in the retina. Our assumption was that Rtbdn might be a key player in sustaining the high levels of flavins in the retina and, thus, supporting retinal metabolic activities. Given the significance of flavins in metabolic processes and the lack of knowledge about how the retina obtains and retains high flavin levels, we assessed the role of Rtbdn in regulating retinal flavins (Kelley et al., 2015). We confirmed the extracellular nature of Rtbdn and its ability to electrostatically bind to the plasma membrane (Kelley et al., 2015). We also found that Rtbdn can bind RF *in vitro* (Kelley et al., 2015) and *in vivo* (Kelley et al., 2017), confirming the function of Rtbdn as an RF carrier between the retina and the RPE.

## 3 The Protective Role of Retbindin

To test the neuroprotective abilities of Rtbdn, 661W cells were exposed to 11,000 lux for 1 hour in the presence of membrane preparations isolated from COS-7 cells either untransfected or transiently transfected with a vector to express Rtbdn or RBP (Kelley et al., 2018). The 661W cells were shown to be from a cone-cell lineage (Tan et al., 2004) and are sensitive to exposure to high light intensities (Kelley et al., 2018). Following light exposure in the absence of Rtbdn-containing membrane preparations, ∼70% of the cells died, while 100% of the cells supplied with Rtbdn-containing membrane preparations survived (Kelley et al., 2018). This level of protection was comparable to cells given 100 nM Docosahexaenoic acid (DHA) in the medium (Kelley et al., 2018), which has been shown to have an anti-apoptotic role in the retina (Querques et al., 2011). Furthermore, supplementation with RBP-containing membrane preparations showed an analogous effect as the supplementation with Rtbdn-containing membrane preparations (Kelley et al., 2018).

Rtbdn ablation led to a reduction in rod and cone functions and a time- and dose-dependent retinal degeneration starting as early as P120 and progressing at P240 (Kelley et al., 2017), providing additional evidence for its protective role. This degenerative process was accompanied by a significant reduction in total retinal flavin levels (Kelley et al., 2017). Subsequently, we showed that the absence of Rtbdn in the knockout mice (Rtbdn^−/−^) and the ensuing reduction in retinal flavins levels led to significant misregulation in the levels of multiple metabolites involved in the citric acid cycle prior to the onset of degeneration (Sinha et al., 2021). Among other metabolic changes, the neural retina of Rtbdn^−/−^ mice displayed a significant reduction in glutathione recycling, accumulation of toxic metabolites, and increased methionine sulfonation, all of which contribute to the degenerative phenotype associated with Rtbdn ablation (Sinha et al., 2021). Sphingolipid metabolism is hampered by the lack of glucose transport through serine production, and toxic hexasylceramide accumulates (Sinha et al., 2021). This causes the neural retina to inhibit glycolysis by inhibiting tetrameric pyruvate kinase M2 (PKM2) formation and instead upregulating the pentose phosphate pathway (PPP) to counteract the oxidative damage. Lipolysis cannot function properly in the presence of diminished flavin cofactors, resulting in acylcarnitine accumulation in the Rtbdn^−/−^ retina. Moreover, as the metabolic load increases, the gap between ATP demand and supply widens, causing the metabolic homeostasis to be disrupted. This is accompanied by a significant decrease in amino acid metabolism in Rtbdn^−/−^ neural retina and RPE (Sinha et al., 2021). The findings from Sinha et al. showed that even small alterations in retinal metabolic homeostasis can shift the balance between a healthy and pathogenic state, ultimately affecting retinal function (Sinha et al., 2021). In fact, we found that ATP levels were reduced in P45 neural retinas in absence of Rtbdn and declined further as the animal aged. Overall, our findings indicate that Rtbdn is an important participant in flavin modulation in the retina and the association between retinal degeneration and the reduction in flavin levels strengthened our hypothesis about the neuroprotective role of Rtbdn.

In a recent publication, Fan et al. (2021) proposed that Rtbdn mediates retinal light damage and has significant retinoid-binding affinity, most notably 11-cis retinal. The authors also claimed that in the absence of Rtbdn, there was no drop in total flavin levels. They based their conclusions on two pieces of evidence. First, they used bio-layer interferometry binding studies to show that Rtbdn has a high binding affinity for retinoids, especially 11-cis retinal. In our studies, we showed that Rtbdn binds RF *in vitro*, *ex vivo*, and *in vivo* systems. The findings of Fan et al. would have been validated should they have performed direct binding assays to confirm the ability of Rtbdn to bind 11-cis retinal or any other retinoids in retinal explants or *in vivo*. Furthermore, it would have been most informative should Fan et al. measured and demonstrated alterations in retinal retinoids in the Rtbdn knockout retina. As far as the levels of flavin in their Rtbdn^−/−^ mouse line, Fan et al. showed a reduction of 12.5% in RF levels, 3% in FAD, and 4.6% in FMN in aphakic eyeballs (Fan et al., 2021), which contains all eye components except the lens. In fact, it is most likely that the changes in retinal flavin levels in their measurements are masked by the significantly higher flavin levels in the cornea (Batey and Eckhert, 1991), RPE (Sinha et al., 2018), iris, and sclera. Interestingly, we showed the RPE contains ∼20 times more flavin levels than the neural retina (Sinha et al., 2018). It is very clear that they underestimated the reduction in retinal levels of flavin in their Rtbdn^−/−^ line. Regardless, in our studies, we measured flavin levels in isolated neural retinas from our Rtbdn^−/−^ line and showed a 50% reduction when compared to age-matched wild-type neural retinas.

## 4 Neuroprotective Effects of Rtbdn in the Retina

To investigate the neuroprotective effects of Rtbdn, its levels were determined in mouse models of retinal degeneration (Rho^P23H/+^ and Prph2^R172W^). Furthermore, Rtbdn was ablated in these models, and the change in the rate of retinal degeneration was determined. Table 1 summarizes the retinal phenotypes observed in these models.

**TABLE 1:** Inherited retinal degenerative mouse models used in the study of the neuroprotective effects of Rtbdn.

Mouse Model	Prph2^R172W^	Rho^P23H/+^	Prph2^Y141C/+^
Type of mutation	In codon 172 of the Prph2 gene	In codon 23 of the Rho gene	In codon 141 of the Prph2 gene
Clinical phenotype in patients	Cone-rod dystrophy, reduced central vision, and night blindness	Retinitis pigmentosa and night blindness	Pattern dystrophy, macular changes, RPE pigmentation, drusen-like deposits, and chorioretinal atrophy
Mouse model genetic construction	Transgenic	Knock-in	Knock-in
Retinal phenotype in mice	Cone loss followed by secondary rod loss	Rod loss followed by gradual cone death	Rod and cone death and accumulation of yellow flecks in the RPE
Cell type affected	Cone first, then rods	Rods first, then cones	Rods and cones
Mutant protein fate	The R172W mutation led to a conformational shift in the structure of the mutant protein that negatively affected cones more than rods	A small fraction of P23H protein (1–10%) traffic to the OS	The addition of extra cysteine in the Prph2-Y141C led to the formation of high molecular weight complexes that was able to initiate OS formation but not proper disc growth
Source	Ding et al. (2004)	Sakami et al. (2014)	Stuck et al. (2014)

### 4.1 Elimination of Rtbdn Exacerbates Disease Progression in the Rho^P23H^ Knockin Model of Retinitis Pigmentosa

Retinitis pigmentosa (RP) represents inherited retinal dystrophy caused by the progressive loss of photoreceptors, with no effective therapy available thus far. RP affects over 1.5 million patients worldwide, with a prevalence of 1:4,000 (reviewed by Verbakel et al. (2018)). Therefore, identifying the factors that maintain retinal homeostasis and promote photoreceptor survival during the pathogenesis of RP is critical for the treatment of this disease. In most cases of RP, the rod photoreceptors in the peripheral retina begin to degenerate first with a gradual development of night blindness (Hartong et al., 2006). With the increasing loss of rods, the cones gradually die, ultimately resulting in complete blindness, making it more challenging to develop treatment strategies. Although the pathogenesis of RP is still not clear, recent studies have found oxidative stress build-up during rod and cone degeneration in RP (Moreno et al., 2018; Trachsel-Moncho et al., 2018; Newton and Megaw, 2020).

Around 25% of all RP cases are caused by mutations in the rhodopsin gene (RHO) (Hartong et al., 2006), with a proline to histidine change at codon 23 (P23H) in RHO being the first RP mutation identified in human patients (Dryja et al., 1990a; Dryja et al., 1990b). In addition, this mutation also represents the most common cause of Rho-related RP in the US (Sung et al., 1991; Sohocki et al., 2001). Various transgenic and knockin animal models have been used for the investigation of the effects of the P23H mutation (Olsson et al., 1992; Naash et al., 1993; Machida et al., 2000; Ross et al., 2012; LaVail et al., 2018). The most widely used models that mimicked the patient’s phenotype and disease progression are several P23H transgenic rat lines (LaVail et al., 2018), a P23H transgenic mouse line (Naash et al., 1993), and a P23H knock-in mouse line (Rho^P23H/+^) (Sakami et al., 2011). The mouse models showed severe functional impairments in rods and less functional deficits in cones at an early age, but cone abnormalities become more prominent at about P60 (Naash et al., 1993; Sakami et al., 2011).

**FIGURE 2:**
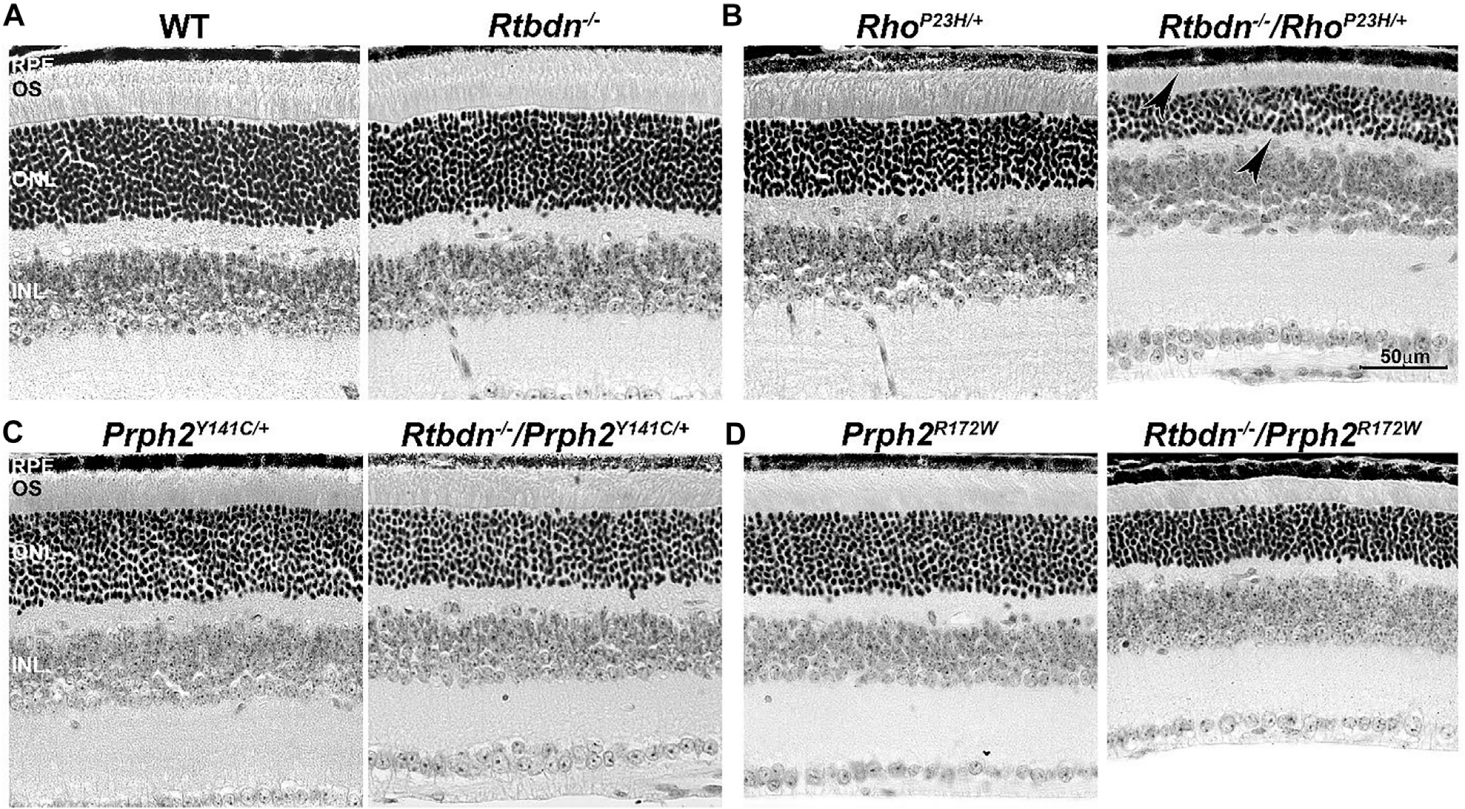
Ablation of Rtbdn accelerates retinal phenotype in several models of degeneration. Histologic analysis at the light microscopic level of retinal sections from WT and Rtbdn^−/−^ **(A)**, Rho^P23H/+^ and Rtbdn^−/−^/Rho^P23H/+^ **(B)**, Prph^Y141C/+^ and Rtbdn^−/−^/Prph2^Y141C/+^ **(C)**, and Prph^R172W^ and Rtbdn^−/−^/Prph2^R172W^ **(D)** mice at P30. Arrowheads point to the degenerated ONL and outer segments shortening in the Rtbdn^−/−^/Rho^P23H/+^ model. Scale bar: 50 µm. RPE, retinal pigment epithelium; OS, outer segment; ONL, outer nuclear layer; INL, inner nuclear layer. Images were taken from paraffin-embedded eyes of the relevant genotypes used by Genc et al. (2020a) and Genc et al. (2020b).

**FIGURE 3:**
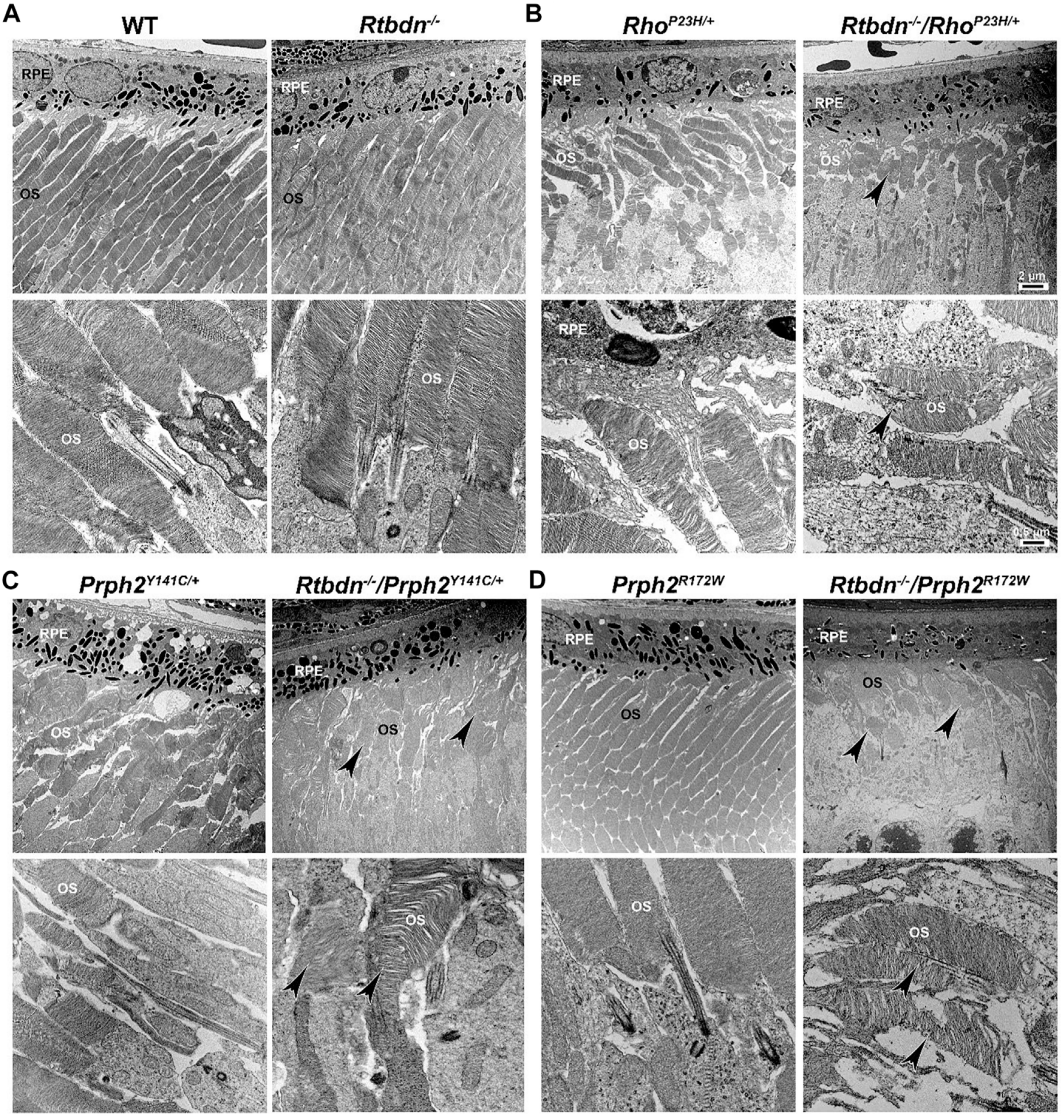
Ablation of Rtbdn exacerbated the changes in outer segment ultrastructure in models of retinal degeneration. Effects of Rtbdn ablation at the ultrastructural level in the retinas of disease models. **(A–D)** Representative TEM images of RPE and OS at two magnifications (5,000× for the upper image and 25,000×for the lower image for each genotype) are presented for the indicated genotypes at P30. Arrowheads point to the shorter and misoriented OSs discs, disorganized and misaligned, or abnormal membrane accumulation when Rtbdn is ablated. Scale bar: 2 µm **(A)**, 0.5 µm **(B)**. RPE, retinal pigment epithelium; OS, outer segment. Images were taken from paraffin-embedded eyes of the relevant genotypes used by Genc et al. (2020a) and Genc et al. (2020b).

To evaluate the role of Rtbdn in retinal degenerative disorders, Rtbdn levels were measured in the retinas of Rho^P23H/+^ mice at P15 (before the onset of degeneration), P30 (left image in Figure 2B, at the onset of degeneration), and P60 (after the onset of degeneration) (Genc et al., 2020b). In their study, Genc et al. showed significant upregulation of Rtbdn levels prior to and during the degenerative process in the Rho^P23H/+^ mice (Genc et al., 2020b). Although the pattern of Rtbdn distribution in the Rho^P23H/+^ retina was still preserved like the wild-type around the inner segment and at the tips of the OS/RPE junction, abnormally large amounts of Rtbdn staining were also seen throughout the OS layer in patches. The identity of these patches is still unknown. Since Rtbdn binds RF and flavins are necessary for a variety of metabolic processes, it is no surprise that Rtbdn is overexpressed before the onset of degeneration and during cell death. This strongly suggests that Rtbdn plays a major role in the cellular stress response, especially knowing that apoptosis requires energy. Notably, the elimination of Rtbdn in the Rho^P23H/+^ retina at P30 caused a significant reduction in both scotopic a- and photopic b-waves amplitudes (Genc et al., 2020b). Structurally, ablation of Rtbdn in Rho^P23H/+^ animals (Rho^P23H/+^/Rtbdn^−/−^) accelerated the onset of degeneration (see arrowheads in Figure 2B and Figure 3B right images) and increased the progression of the disease phenotype (Genc et al., 2020b), despite the fact that there was no apparent phenotype in the Rtbdn^−/−^ retinas until P120 (Kelley et al., 2017; Genc et al., 2020b). When Rtbdn was eliminated, there were no initial changes in flavin levels at P15 (Genc et al., 2020b), but those levels were reduced in older Rho^P23H/+^ retinas despite Rtbdn upregulation. This could be the outcome of excessive flavin consumption by the surviving cells or due to reduced delivery by the RPE, which undergoes stress due to increased cellular debris from dying photoreceptor cells and/or changes in its metabolic activity as a result of the metabolic alterations occurring in the neural retina (Sinha et al., 2021) or a decrease in the number of surviving photoreceptor cells (Genc et al., 2020b).

### 4.2 Elimination of Rtbdn Worsens the Disease Progression in the Prph2^Y141C^ Model of Pattern Dystrophy

The tetraspanin protein Prph2 is a photoreceptor-specific membrane protein that is required for the morphogenesis of both rod and cone OSs rims (Sanyal and Jansen, 1981; Connell et al., 1991; Travis et al., 1991; Stuck et al., 2016). Mutations in the PRPH2 gene are associated with a variety of pathologic conditions, including central areolar choroidal dystrophy, autosomal dominant retinitis pigmentosa, various forms of macular degeneration, and pattern dystrophy mimicking Stargardt/fundus flavimaculatus (Boon et al., 2008; Reeves et al., 2020). Pattern dystrophy (PD) is a dominantly inherited macular disease characterized by lipofuscin buildup in the RPE. Mutations in the PRPH2 gene have been identified to associate with or cause different forms of PD at different times of onset (Francis et al., 2005). It has been hypothesized that mutant PRPH2 leads to PD by disrupting the integrity of the photoreceptor disc membrane, resulting in photoreceptor cell death and accumulation of lipofuscin in the RPE (Francis et al., 2005). One of the mutations in PRPH2 that leads to PD is the Y141C, which is dominantly inherited (Yang et al., 2004). A knockin mouse line for this mutation was generated and showed a retinal phenotype similar to that seen in patients carrying the Y141C mutation (Stuck et al., 2014). Histological assessments of the heterozygous retinas taken from P30 Prph2^Y141C/+^mice revealed a reduction of about two rows of photoreceptor nuclei (Figure 2C, left image) with significantly shorter OSs (Figure 3C, left images) (Stuck et al., 2014). The Prph2^Y141C/+^ knock-in mouse model shows impaired rod and cone function, progressive retinal degeneration, and broad diffused yellow flecks in fundus imaging (Stuck et al., 2014).

Similar to Rho^P23H/+^, Rtbdn levels are significantly increased in Prph2^Y141C/+^ retinas compared to WT retinas, and ablation of Rtbdn accelerated the degenerative process (Genc et al., 2020b). Prph2^Y141C/+^/Rtbdn^−/−^ exhibited reduced scotopic a- and photopic b-waves amplitudes and ultrastructural defects (see right images in Figure 2C and Figure 3C) (Genc et al., 2020b). Prph2^Y141C/+^/Rtbdn^−/−^ OSs were also very short and round and exhibited whorl formation (Figure 3C, right images) (Genc et al., 2020b).

The abovementioned findings indicate that Rtbdn plays a protective role for both rods and cones during photoreceptor degeneration, even though Rtbdn is only expressed by rods (Kelley et al., 2015). Rtbdn is likely to be required for retinal homeostasis because it is involved in the enrichment of retinal flavins by extracellularly binding to RF and potentially increasing the active concentration of RF in the extracellular environment (Genc et al., 2020b).

### 4.3 Elimination of Rtbdn Exacerbates Disease Progression in the Prph2^R172W^ Model of Cone-Rod Dystrophy

One of the most common mutations in PRPH2 is at codon 172, where arginine is substituted by tryptophan and is observed in patients diagnosed with either retinitis pigmentosa or macular dystrophy (Wells et al., 1993). This led to the conclusion that the function of certain domains in PRPH2 may be different in cones and rods. A transgenic mouse model for this mutation (Prph2^R172W^) was generated and showed a retinal phenotype similar to that of the patients (Ding et al., 2004). The R172W animals showed late-onset degeneration that became initially apparent in cones, followed by degeneration of the rods (Ding et al., 2004), consistent with what has been seen in patients affected by this mutation. No significant degeneration is seen in the P30 Prph2^R172W^ retina (Figure 2D, left image) with the normal ultrastructural organization of the discs (Figure 3D, left images).

Genc et al. investigated Rtbdn’s levels in retinal extracts from Prph2^R172W^ mice and reported that Rtbdn levels were significantly upregulated before (at P30) and during (P90) retinal degeneration (Genc et al., 2020a). Again, the absence of Rtbdn (Prph2^R172W^/Rtbdn^−/−^) exacerbated functional and structural decline (see right images in Figure 2D and Figure 3D) accompanied by retinal gliosis (Genc et al., 2020a). Overall, the Prph2^R172W^/Rtbdn^−/−^ mice displayed a significantly more severe phenotype than either Rtbdn^−/−^ or Prph2^R172W^ mice (Genc et al., 2020a).

## 5 Future Perspectives

IRDs are a diverse set of hereditary abnormalities that affect the retina and lead to photoreceptor malfunction and vision loss. Treatments for IRDs are currently unavailable, but new options are rapidly evolving. Advances in vectors (viral and non-viral) and delivery systems to carry therapeutic genes to the retina are very promising. These vectors can deliver the normal gene in cases where gene augmentation is sufficient or neuroprotective genes. Delivery of a specific gene will only provide therapy for those IRDs caused by that mutant gene; however, delivery of a neuroprotective gene allows for a therapy that spans many different IRDs. Therefore, the search for neuroprotective genes is critical for the accomplishment of this goal. We found that the RF-binding protein, Rtbdn, is significantly increased during retinal degeneration, irrespective of the causative mutation or mutant gene. Furthermore, we have shown that ablation of Rtbdn exacerbates the degenerative process. Finally, the addition of Rtbdn-containing membrane preparations to the 661W cells, which are known to be light-sensitive, protected these cells from light-induced cell death. Combined, these data support a protective role for Rtbdn in the retina, making it a promising candidate in the development of a pan-mutant therapy.

Going forward, Rtbdn expressing vectors packaged into non-viral nanoparticles will be used in gene therapy experiments in mouse models of retinal degenerative diseases. Since Rtbdn is extracellular in nature, it will be interesting to investigate whether targeting Rtbdn expression to the RPE is as effective as targeting it to the neural retina. This strategy is motivated by the RPE’s ability to take up therapeutic vectors much more readily than the neural retina. Furthermore, delivery to the RPE can be achieved *via* suprachoroidal means, which prevents the retinal detachment that occurs with subretinal delivery. We have previously shown that some proteins present in the retina are expressed by the RPE (Kanan et al., 2009). In addition, we believe that Rtbdn is an ideal therapeutic target due to its localization at the OS tips, critical for the interrelated functions of the RPE and retina, and given that many IRDs have secondary RPE and choroidal defects.

To summarize, future investigations on Rtbdn and the mechanism of flavin regulation in the retina are needed to establish how it exerts its neuroprotective effects. However, that should not prevent attempts to determine the protective effects of Rtbdn through gene therapy approaches.
